# CSA@g-C_3_N_4_ as a novel, robust and efficient catalyst with excellent performance for the synthesis of 4H-chromenes derivatives

**DOI:** 10.1038/s41598-023-46122-y

**Published:** 2023-11-03

**Authors:** Saber Hosseini, Najmedin Azizi

**Affiliations:** Chemistry and Chemical Engineering Research Center of Iran, P.O. Box 14335-186, Tehran, Iran

**Keywords:** Catalysis, Green chemistry, Organic chemistry

## Abstract

A pioneering robust and green heterogeneous acidic catalyst (CSA@g-C_3_N_4_) was rationally designed via immobilization of camphorsulfonic acid (CSA) on the g-C_3_N_4_ surface under mild conditions. Grafting CSA in the g-C_3_N_4_ lattice is distinguished as the root cause of facilitating the structure change of g-C_3_N_4_, leading to a unique morphology, accordingly the remarkable catalytic efficiency of CSA@g-C_3_N_4_. The morphology of new as-prepared nano-catalyst was specified by means of FT-IR, XRD, SEM, EDS, TEM, TGA, and BET. For the first time, it is exhibited that the efficient catalyst CSA@g-C_3_N_4_ can productively accomplish the three-component reactions with high yields and also serve as an inspiration for easily performing various sorts of MCRs based on our finding. The recommended synthesis pathway of chromenes derivatives is facile and cost-effective which applies a condensation reaction of salicylaldehyde, thiophenol, and malononitrile followed by ready purification in a benign manner. Moreover, the CSA@g-C_3_N_4_ nanocomposite can be promptly reused, illustrating no sensational decrease in the catalytic activity after ten times.

## Introduction

Today, solid acid catalysts have earned pervasive notice in organic synthesis as well as transformation. Since, they are non-toxic, chemically stable, non-corrosive, economical, highly selective, considerably active, and reusable with no depletion in activity, which can also be easily operated under the mild reaction conditions followed by a simple separation process^1–3^. Moreover, acidity classification either Brønsted or Lewis, the acidic site potency and accessibility, alongside support specification concerning porosity, surface area, and thermal and chemical stability, are counted as key parameters affecting the solid acid catalysts traits and efficiencies. Thereby, the performance of the catalysts can be promoted via exploiting an appropriate support^4, 5^.

More specially, the benign and green catalyst of graphite-like carbon nitride (g-C_3_N_4_) is of extensive attention due to some potential attributes^6–11^. In addition, g-C_3_N_4_ seems to be a prime candidate in selective organic transformation as catalysts ascribed to its exclusive features and low cost^12–17^. A satisfactory approach to increase the surface area would be the fabrication of mesoporous carbon nitride via assigning pores within the scaffold. This happens by employing various types of templates in which emerging number of problems is inevitable. In other words, withdrawing hard templates cause non-environmental attributes, and deploying soft templates leads to undesired residuals into the g-C_3_N_4_ construction^18–22^. In other trials by scientists, porous carbon nitrides with the escalated surface area have been manufactured without utilizing templates as outlined in the following. For that reason, acid treatment of the bulk g-C_3_N_4_ or even consuming other materials, precursors pretreatment as another procedure have become promising trends for creating porous structure^23–27^. Notably, multi polyhydroxyl arrangements of the sulfonic groups could adjust spreading the electrons on the surface of the catalyst, which expedites the half-reactions of electrons^28^. On the side of recent concerns regarding environmental issues, metal-free organocatalysts can be the opportune recommendation. Another fascinating substance for being applied in several organic transformations, including multicomponent reactions, is camphorsulfonic acid (CSA) as a productive, non-hazardous, affordable, benign, commercially accessible, and soluble in water Brønsted acid catalyst^29, 30^.

Modern organic chemistry has put a specific focus on identifying new synthetic procedures to expedite the formation of organic compounds. To meet this challenge, the establishment of multicomponent reactions (MCRs) is regarded as a worthwhile approach applied to the production of complex molecules through executing several stages in a single process^31, 32^. Accordingly, MCRs play a leading role in the synthesis of various key heterocyclic compounds, for example, chromenes derivatives. Additionally, chromene moiety is frequently spotted as a crucial constructional unit either in biologically active or natural materials, such as flavonoids, alkaloids, anthocyanins plus tocopherols. More precisely, one of the most critical uses of functionally substituted chromene can be synthesizing compounds with the potential to be exploited in the medicinal chemistry branch^33–41^.

The application of solid acid catalysts in various areas has grabbed scientists’ attention in recent years as if it has become an attractive field^42–44^. On the other hand, the synthesis of chromene compounds employing diverse methodologies, including the use of acid catalysts or other methods, has been discussed in various articles in recent years. Hence, these reports have tried to express that developing a new reaction route for fabricating novel chemical compounds with different molecular constructions is of remarkable value in chemistry plus biology areas. Accordingly, developing approaches in which cost-effective catalysts along with more moderate conditions of reaction can be exploited has grabbed ascending attention in synthesizing structurally different frameworks. In this context, Lewis acids such as Al(OTf)_3_ or FeCl_3_ and a less toxic solid acid *p*-TSA catalyst as a Brønsted Acid have been used for the chemoselective synthesis of 4H-chromenes. In a comparison based on the recorded reports, it can be concluded that, in general, the use of Brønsted acids as a catalyst rather than Lewis acids increases the yield of products in addition to a significant decrease in the reaction time for chromene production^45–47^. One of the recently mentioned solid acid catalysts is MNPs•GO-CysA, which has been effective in the one-pot synthesis of 4H-chromene scaffolds from three components^48^. In this regard, nanostructured carbon compounds have raised tremendous interest in authentic documents around nanomaterials owing to promising qualities, such as low price, air stability, and corrosion resistance to be deployed in various chemical as well as industrial operations. Among other catalysts from a category of carbon compounds (graphene oxide) that have been explored for synthesizing 4H-chromene derivatives, Fe_3_O_4_‐supported sulfonated graphene oxide and the piperazine immobilized on the graphene oxide surface can be pointed out^49, 50^. Among other classes of new catalysts for chromene synthesis, MOFs should also be taken into consideration. Furthermore, heterogeneous solid acid catalyst systems, on the basis of metal organic frameworks, could be achieved by introducing acid sites on the MOF surface via chemical modifications, for example, Fe_3_O_4_@UiO@DAS^51^. In another case, the use of various Fe_3_O_4_-based hydrotalcites acting as acid–base bifunctional catalysts was stated to synthesize different 4H-chromene derivatives by a three-component reaction^52^. To gain the potential advantages of applying multifunctional catalysts in MCRs along with designing productive and green approaches in organic synthesis as a part of our research prospect, it can even be referred to the reports in which methods other than utilizing solid acid catalysts have been used for chromene synthesis. In this framework, agricultural wastes as an alternative source for toxic and dangerous catalysts for the environment have shown the capability to serve as green catalysts due to their ease of biodegradability. Moreover, water extract of pomegranate peel ash (WEPPA), water extract of lemon fruit shell ash (WELFSA), and Water extract of mango peel ash (WEMPA) are some examples of natural aqueous medium catalysts that have participated in producing 4H-chromene derivatives through the multicomponent reactions under exposure to microwave in recent years^53–55^. In an engaging report, multi-component reactions in combination with photocatalytic processes for 4H-chromene synthesizing have been investigated^56^. Additionally, green electrocatalytic multicomponent synthesis has been studied as another technique of 4H-chromenes production, exploiting potassium iodide as an electrolyte in undivided cell, which production yields of 86% have been reported^57^. Furthermore, ionic liquids have demonstrated an extensive domain of applications as either promoters or reaction medium in the case of multi-component reactions. In the last few years, a number of procedures employing various sorts of ionic liquids have been introduced to gain 4H-chromene derivatives^58^. Multicomponent reactions (MCR) provision deep knowledge regarding 4H-chromene formation. Furthermore, considerable catalytic schemes have evolved over recent years for the multicomponent synthesis of 4H-chromene derivatives, including RGO-SO_3_H^59^, GO/α-Fe_2_O_3_/CuL^60^, MNPs@Cu^61^, g-C_3_N_4_^62^, Pd@g-C_3_N_4_^63^, CaMgFe_2_O_4_^64^, morpholine^65^, CAEDA^66^, mica/Fe_3_O_4_^67^, amino grafted MOFs^68^, DABCO^69^, Fe_3_O_4_@Sal@Cu^70^, Fe_3_O_4_@GO-NH^71^, KCC-1@NH_2_^72^, Nano-SiO_2_^73^, SnO_2_ NPs^74^, Ag_2_O/GO/TiO_2_^75^, and borax^76^. Yet, there are several drawbacks concerning plenty of expressed catalysts, including environmental contamination, high price, challenging catalyst removal, and requiring coarse reaction conditions. There are still tremendous requests for designing more practical, facile, benign, and efficient methods to synthesize these classifications of heterocyclic compounds ascribed to their prominence and expansive application.

As-mentioned valuable traits of chromenes derivatives act as incentives to develop productive and environmentally friendly procedures for 4H-chromenes framework synthesis employing CSA@g-C_3_N_4_. We have been prompted to prepare a novel heterogeneous catalyst via surface modification of g-C_3_N_4_ using camphorsulfonic acid for the first time. This protocol supports the impacts of green chemistry in synthesizing the chromenes derivatives through a three-component reaction while utilizing acid to accelerate catalytic activity at the same time.

## Result and discussion

Extraordinary surface tailoring of g-C_3_N_4_ was effectively achieved through the embedding of camphorsulfonic acid (CSA) to produce CSA@g-C_3_N_4_. This fruitful synthesized catalyst maps out new routes toward the progressions of forthright synthesis of 4H-chromenes derivatives. Nano-carbon nitride-supported camphorsulfonic acid as a unique catalyst was designed and fabricated in the convenient pathway delineated in Figure S1 (see details in the Figure S1). The catalyst of CSA@g-C_3_N_4_ was characterized via various methods, namely FT-IR, XRD, SEM, EDS, TEM, TGA, and BET.

### Characterization of synthesized CSA@g-C_3_N_4_

#### Fourier transform infrared spectroscopy (FT-IR)

**Figure 1:**
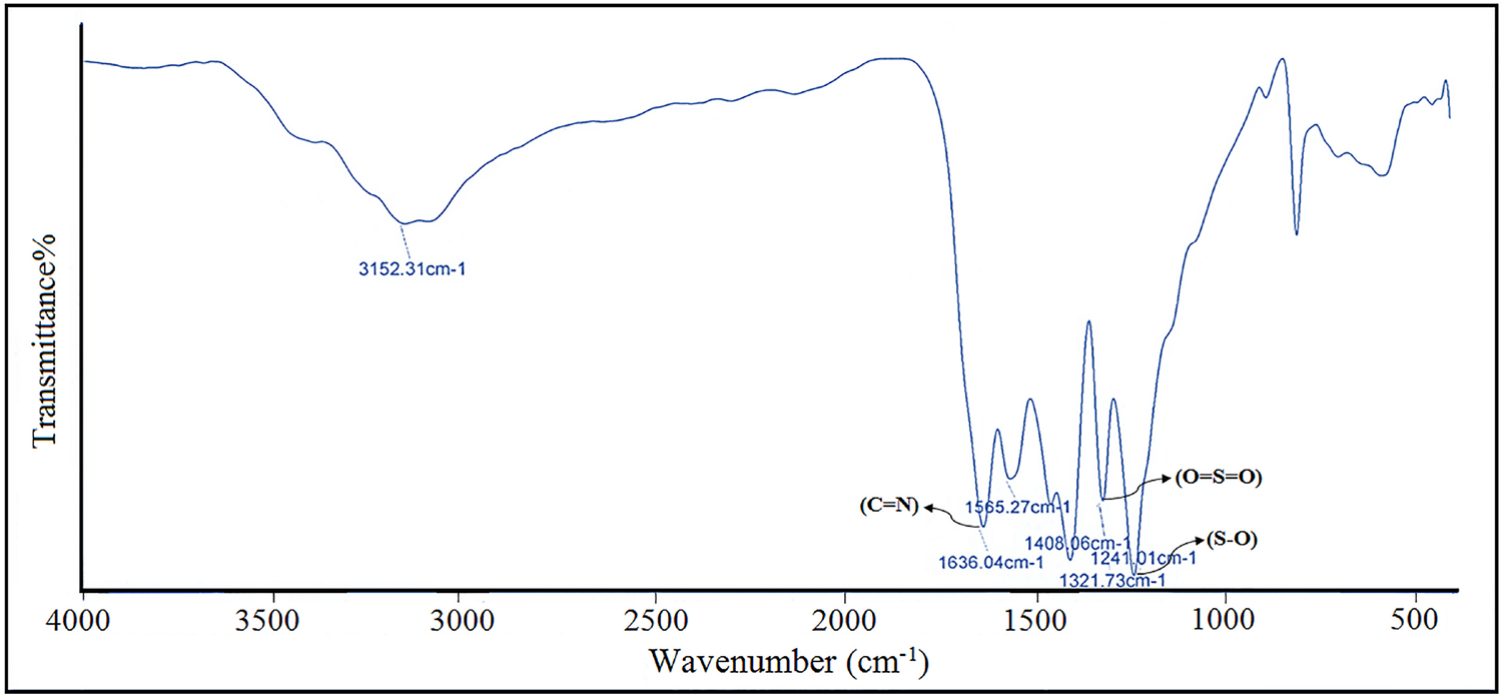
The FT-IR spectrum of CSA@g-C_3_N_4_.

FT-IR (Fourier transform infrared spectroscopy) was assisted to indicate the functional groups, construction added to chemical bonds vibration of the as-prepared catalyst depicted in Fig. 1. The broad bands nearby 3000 to 3500 cm^−1^ mostly reveals the NH stretching vibrational modes associated with NH_2_ groups belong to g-C_3_N_4_ in addition to O–H bands attributed to the adsorbed water on the surface of catalyst^77^. The heterocyclic stretching vibration of C=N was detected by absorption band located at the area close to 1636 cm^−1^. The absorption bands at the 1408–1565 cm^−1^ zone were corresponded to C-N vibrational spectra of nitrogen-entailing aromatic rings (N–C=N) regarding to s-triazine as well as tri-s-triazine which are existed in the g-C_3_N_4_. Moreover, manufacturing of g-C_3_N_4_ from heptazine blocks was evidently approved by a sharp peak at 806 cm^−1^ assigned to heptazine rings bending vibration^78^. The apparent bands at 1241 and 1321 cm^−1^ allocated to S–O and O=S=O stretching vibration, respectively, in the FT-IR spectrum imply that the g-C_3_N_4_ encompasses –SO_3_H groups. The appearance of NH, acidic SO_3_H together with absorbed water was confirmed via wide stretching absorption placed in the area of 2100–3000 cm^−1^^79^. Ascribed to the FT-IR spectrum of nano-CSA@g-C_3_N_4_ specifies the existence of the camphorsulfonic acid on the surface of pure g-C_3_N_4_.

#### X-ray powder diffraction (XRD)

**Figure 2:**
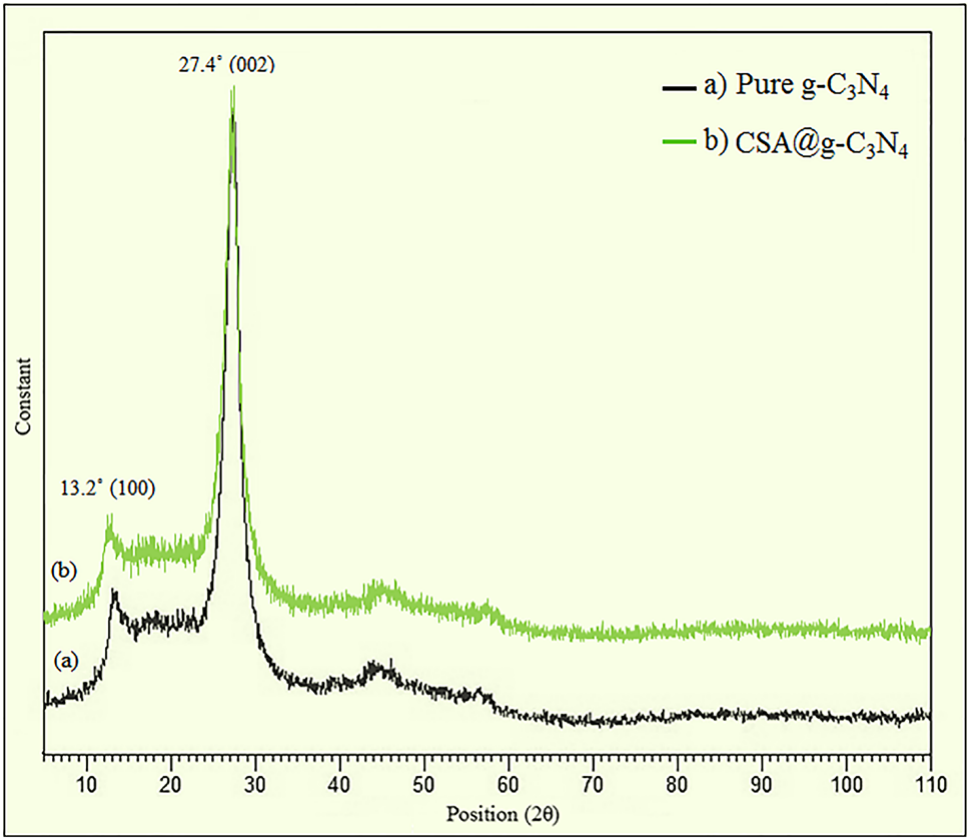
The X-ray diffraction patterns of the (**a**) pure g-C_3_N_4_ and (**b**) CSA@g-C_3_N_4_.

A large-angle x-ray powder diffraction (XRD) technique is a tool for inspecting the impact of -SO_3_H groups on the g-C_3_N_4_ crystalline features. Figure 2 depicts the XRD patterns of g-C_3_N_4_ before and after modification by camphorsulfonic acid. The crystalline nature of the carbon nitride polymer was ascertained through two peak intensities. The main one with high intensity was marked at 2θ = 27.4° (002) (JCPDS card No. 87-1526) attached to the s-triazine units interplanar stacking ordering. On the other side, the diffraction characteristic peak with low intensity at 2θ = 13.2° (100) illustrated the attendance of graphite-resembling interlayer structural packing of aromatic heterocyclic units^80, 81^. Additionally, the similarity between the crystalline peaks of pure g-C_3_N_4_ and CSA@g-C_3_N_4_ affirmed the crystalline phase stability of g-C_3_N_4_ showing no destructive effect throughout surface modification process by camphorsulfonic acid. More emphatically, by modifying the surface of g-C_3_N_4_ and subsequently changing the morphology, emerging a strong XRD peak intensity was not for beyond the prediction.

#### Scanning electron microscopy (SEM)

**Figure 3:**
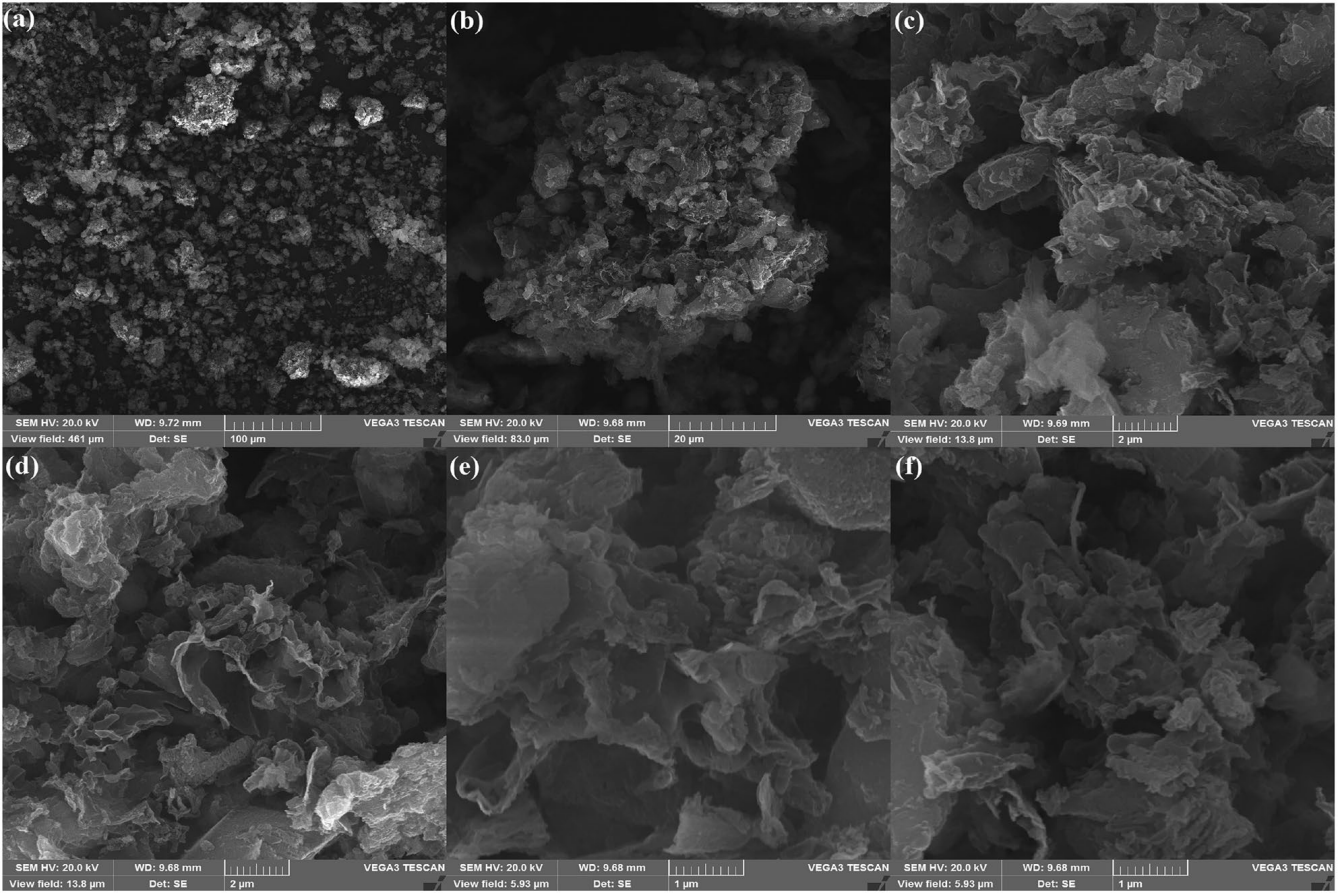
SEM images of CSA@g-C_3_N_4_ with scale bar of (**a**) 100 µm, (**b**) 20 µm, (**c**, **d**) 2 µm, and (**e**, **f**) 1 µm.

The scanning electron microscopy (SEM) images of CSA@g-C_3_N_4_ nano-catalyst to evaluate surface morphology plus distinguishing the shape as well as size of particles is shown in Fig. 3^82^. The as-prepared catalyst nano-scale was clarified owing to the images gathered from SEM. A layered, even, sheet-resembling architecture with irregular configuration could be displayed which agglomeration of some particles were occurred, according to Fig. 3. Further, noticeable alteration in the both size and shape of the g-C_3_N_4_ did not observed after embedding camphorsulfonic acid on the surface of g-C_3_N_4_.

#### Energy dispersive X-ray (EDX)

The energy-dispersive X-ray spectroscopy (EDX) was employed to analyze the elemental distribution or chemical specification of the synthesized catalyst^83^. Fig. S2 displays the EDS analysis of CSA@g-C_3_N_4_. The EDX spectrum indicated the attendance of carbon, nitrogen, oxygen plus sulfur which certifies the successful grafting of camphorsulfonic acid on the surface of g-C_3_N_4_ (see details in the Figure S2).

#### Transmission electron microscopy (TEM)

**Figure 4:**
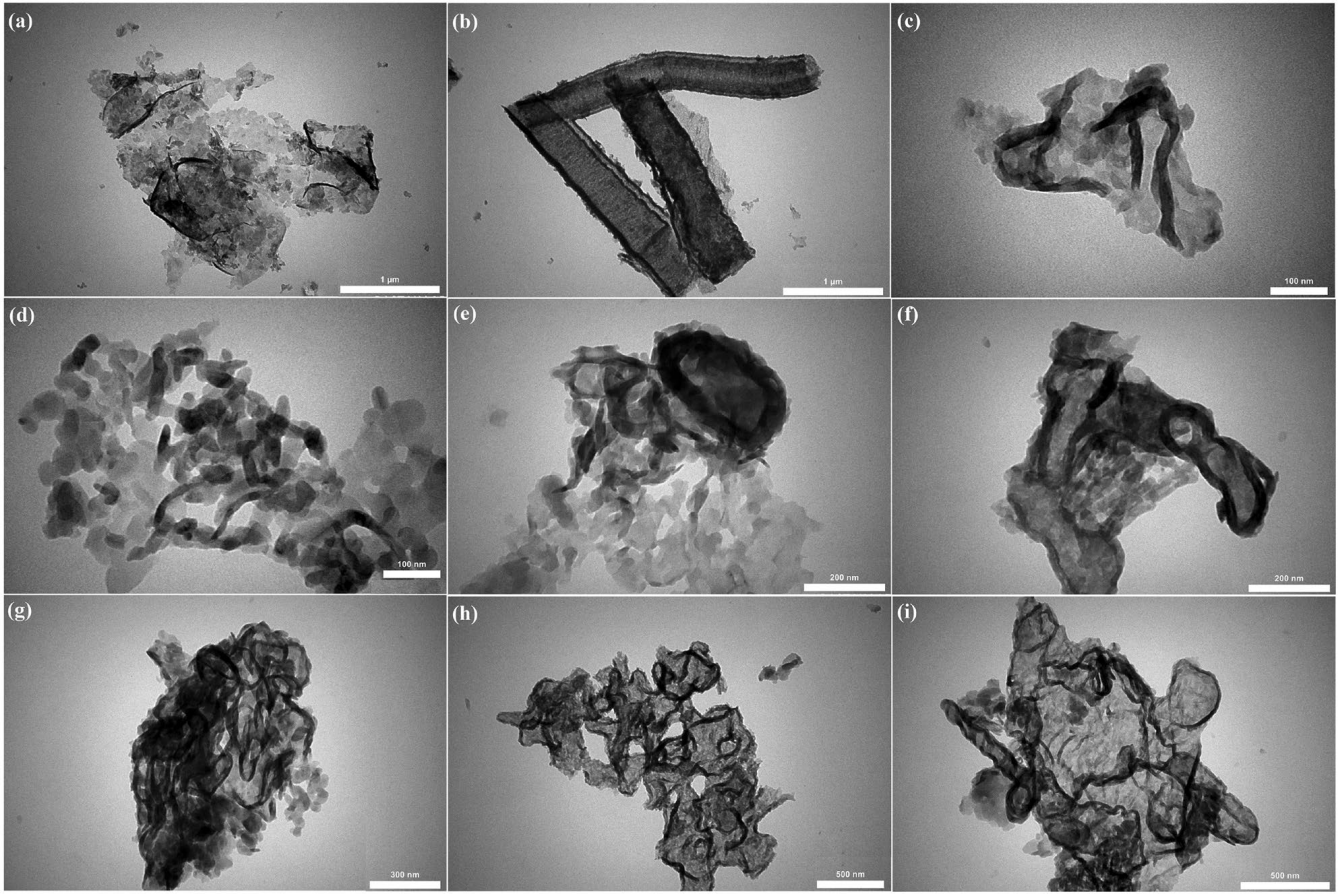
TEM images of CSA@g-C_3_N_4_ with scale bar of (**a**, **b**) 1 µm, (**c**, **d**) 100 nm, (**e**, **f**) 200 nm, (**g**) 300 nm, and (**h**, **i**) 500 nm.

Transmission electron microscopy (TEM) was exploited to gain supplementary confirmation for the morphology in addition to the size distribution of the nano-catalyst. Figure 4 displays the transmission electron microscopy (TEM) images corresponding to the CSA@g-C_3_N_4_ for assessing its surface morphology. Moreover, the layered, even, sheet-like framework with its irregular structure was observed and the agglomeration of some particles were also occurred. Accordingly, the acquired results were satisfactorily consistent with SEM images.

#### The thermo gravimetric analysis (TGA)

**Figure 5:**
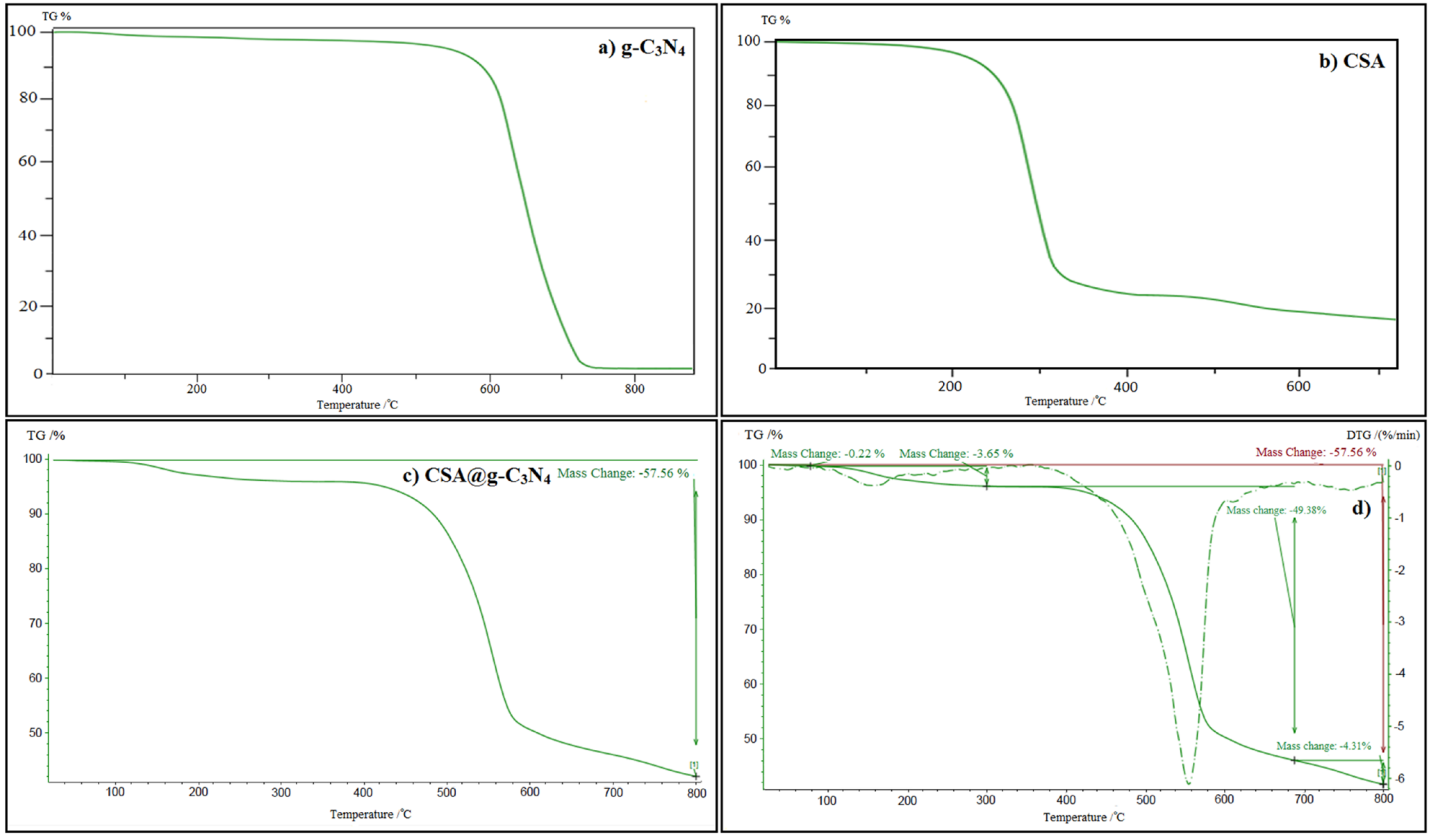
TGA curve of (**a**) g-C_3_N_4_, (**b**) CSA, (**c**) CSA@g-C_3_N_4_ and (**d**) DTG of CSA@g-C_3_N_4_.

Thermal gravimetric analysis (TGA) in parallel with derivative thermogravimetry (DTG) were performed to assess the thermal characteristics of the incorporation of camphorsulfonic acid on the catalyst surface. TGA curves corresponding to bare CSA and pure g-C_3_N_4_ with the as-prepared catalyst of CSA@g-C_3_N_4_ are exhibited in Fig. 5. The TGA curves were taken under temperature up to 800 °C with steady heating rate of 10 °C min^−1^ undergoing air atmosphere as portrayed in Fig. 5. TGA thermographs exhibited the occurrence of several phase transformations as well as transforming reactions across such procedures. In the first stage at the temperature less than 200 °C, the removal of some of those organic compounds exploited in the production process of catalyst or absorbed water stirred up a scant mass loss. Besides, the weight of the nano-catalyst quickly declined in the temperature ranged from 125 to 440 °C owing to the remaining polyhydroxy components in the CSA@g-C_3_N_4_. Moreover, the significant endothermal peak could be seen in the range of 150 to 500 °C owing to the -SO_3_H groups thermal decomposition, which leads to the mild decline in weight, equals to 3.65%. A mild descending in weight was occurred in a temperature ranged from 500 to 800 °C rooted in degradation of the CSA@g-C_3_N_4_ lattice. In agreement with TGA results, the notable thermal stability of CSA@g-C_3_N_4_ was specified which vividly depicts its safe application in organic reactions when the temperature is high^84^. According to the literature^85, 86^, the content of CSA on the g-C_3_N_4_ can be calculated via the TGA curve. As can be seen, 0.22% of primary weight loss is related to the loss of physically adsorbed water. In the following, the second weight change of 3.65 wt% is relevant to the slow mass loss of -SO_3_H groups, which exist in CSA. Subsequently, because of the high temperature, g-C_3_N_4_ was totally decomposed or sublimated; hence, the mass percentage reached 53.69% at this stage. It can be deduced that the remaining compound was CSA, and a value of 42.44% was observed for the residual weight fraction without accounting the -SO_3_H groups. Therefore, it can be concluded that the CSA amount in the porous CSA@g-C_3_N_4_ nanocomposites would be equal to 46.09%.

#### The Brunauer–Emmett–Teller (BET)

The Brunauer–Emmett–Teller (BET) surface area (*S*_BET_) of g-C_3_N_4_ and as-prepared catalyst were surveyed^87^. The pore size distribution was valued via the technique of the Barrett–Joyner–Halenda (BJH) in compliance with the nitrogen isotherm adsorption branch. Table S1 outlines the BET surface area together with pore factors related to pure g-C_3_N_4_ and surface engineered catalyst powder acquired in accordance with nitrogen adsorption–desorption isotherm by use of the BJH technique. The more considerable BET surface area for modified catalyst by embedding CSA was attained when compared to the pure g-C_3_N_4_. Moreover, the results demonstrate the greater porosity and pore volume for CSA@g-C_3_N_4_ rather than g-C_3_N_4_ (see details in the Table S1). The immobilization of CSA onto g-C_3_N_4_ scaffold would bring on increasing the surface area along with pore volume for new nano-catalyst. The amplified surface area and pore volume are two impactful parameters on boosting the kinetics of catalytic reaction subsequent to increasing the mass transfer. The higher surface area for the as-prepared catalyst was recognized by BET results rather than the pure g-C_3_N_4_. The increased surface area was an axiomatic justification for enhancing active sites as well as adsorption of the substrate. The modified catalyst meanwhile illustrated escalated catalytic performance due to the expansive specific surface area assisting, effectively conducting the 4H-chromenes formation in a three-component reaction.

#### Surface acidity studies

**Figure 6:**
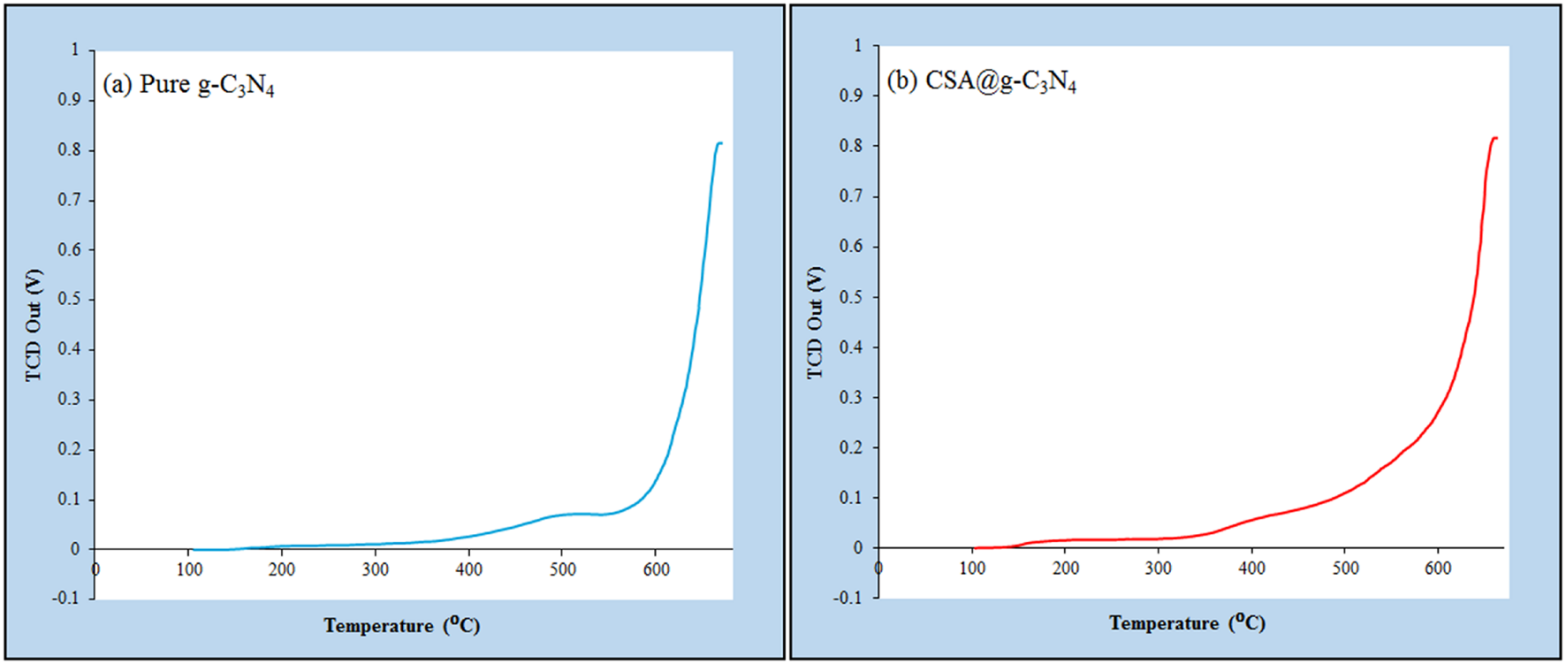
Temperature-programmed desorption of ammonia (NH_3_-TPD) profiles of (**a**) pure g-C_3_N_4_ and (**b**) CSA@g-C_3_N_4_.

To determine the acidity strength of pure g-C_3_N_4_ and CSA@g-C_3_N_4_, the temperature-programmed desorption (TPD) method can efficiently be employed. The basic nature of ammonia is the reason behind selecting an ammonia probe to specify the acidity owing to its facile adsorption on acidic sites by NH_3_-TPD^88–90^. Figure 6 compares the temperature-programmed desorption of ammonia (NH_3_-TPD) profiles corresponding to pure g-C_3_N_4_ and CSA@g-C_3_N_4_ within the temperature scope of 100 to 600 °C. Based on NH_3_-TPD, the weak as well as medium strength acid sites can be revealed in the low temperature between 103 and 300 °C of ammonia desorption, while the high temperatures between 300 and 600 °C in ammonia desorption illustrate the strong acid sites. In fact, by increasing the temperature, the amount of the weak-strength acid sites would decline, while the portion of the medium-strength acid sites would enhance so that strong-strength acid sites would emerge. Furthermore, the TPD profile associated with CSA@g-C_3_N_4_ resembles pure g-C_3_N_4_, showing two differences, reasonably higher intensity together with the more acid sites. Table 1 summarizes the acquired results, portraying the total amounts of acid sites calculated by integrating the NH_3_ desorption peaks. Additionally, the total acidity of the CSA@g-C_3_N_4_ catalyst equals 1124.9 µmol/g, which has dramatically increased compared to the pure g-C_3_N_4_. It should be taken into account that the root cause of the strong Brønsted acidity related to the nano-catalyst of CSA-treated g-C_3_N_4_ is the attendance of considerable density of acid sites on the g-C_3_N_4_ surface brought by -SO_3_H groups, which was certified by the NH_3_-TPD, as demonstrated in Table 1.

**Table 1:** The characteristics of pure g-C_3_N_4_ and CSA@g-C_3_N_4_.

Entry	Catalyst	NH_3_-TPD	
		T (°C)	Acidity amount (µmol/g)
1	g-C_3_N_4_	106–300	40
		300–600	579.6
2	CSA@g-C_3_N_4_	103–300	85.9
		300–600	1124.9

### Evaluation of CSA@g-C_3_N_4_ activity for the synthesis of 4H-chromenes derivatives via the one-step multicomponent approach

A single-pot reaction produces a product via a combination of three reactants or more containing structural features of every applied reagent, all these happen in a process known as MCR. In this work, MCRs merits, such as selectivity and possessing atom economy have been exploited for chromene formation, which plays a leading role in modern synthetic procedures. The topic of this study is the employment of a powerful catalyst for developing MCRs where the absence of a catalyst would trigger not performing the reaction efficiently. According to our captivation in organic synthesis, also being inspired by our previous works focusing on heterocyclic chemistry synthesis; a remarkable output was given, introducing salicylaldehyde as an easy substrate. Therefore, a facile approach was launched in this project for straightforward synthesizing of 4H-chromenes derivatives in a one-pot polymerization, containing salicylaldehyde, malononitrile, various nucleophiles such as thiols, and amines, with employing the catalyst of CSA@g-C_3_N_4_ with pleasant yields under mild conditions at room temperature.

An appropriate support material for non-metal incorporation on the active sites can be graphitic carbon nitride (g-C_3_N_4_), having a configuration similar to graphite to improve the catalytic activity. Accordingly, the CSA doped g-C_3_N_4_ was productively synthesized to upgrade the catalytic performance for three-component reaction in the path of chromene production, decreasing the reaction time, promoting the yield of the reaction, and extending the of ratio product manufactured as well. Along the lines of this strategy, the model reaction between salicylaldehyde, malononitrile, and thiophenol was delved into the assessment of the most optimum reaction condition. It should be note that several solvents such as ethanol (C_2_H_5_OH), dichloromethane (CH_2_Cl_2_), glycerol (C_3_H_8_O_3_), acetonitrile (CH_3_CN), water (H_2_O), ethyl acetate (CH_3_CO_2_Et), tetrahydrofuran (THF), and dimethylformamide (DMF) were utilized in this reaction.

Table 2 assesses the optimum conditions to determine the type as well as amount of catalyst and also the solvent type for achieving the highest efficiency. The pure g-C_3_N_4_ completed the regarded three-component reaction by the yield of 18% to synthesize chromene while CSA performed the reaction alone in 2 h by yield of 71% (Table 2, Entries 13 and 14). The efficient catalyst of CSA@g-C_3_N_4_, which was synthesized for this work, outstandingly elevated the performance of the reaction. Different amounts of catalyst were examined to comprehend the most effective value. When the three-component reaction was conducted by salicylaldehyde, thiophenol and malononitrile without exploiting catalyst, the yield of 25% could be obtained for chromene production (Table 2, Entry 5). Consequently, increasing the amount of catalyst was sensibly enhanced the performance of reaction with respect to results (Table 2, Entries 1–4). To precisely study the reaction on path of seeking the most appropriate conditions, several protic and aprotic solvents were evaluated. The output was identifying ethanol as suitable solvent for three-component reaction of chromene production (Table 2, Entries 1 and 6–12). The optimization results implies that the best efficiency of 92% for chromene synthesis associated with three-component reaction of salicylaldehyde, thiophenol and malononitrile was obtained by deploying 40 mg catalyst of CSA@g-C_3_N_4_ in ethanol (1 mL) as solvent after 2 h at 60 °C.

**Table 2:** Optimization of the reaction conditions in the straightforward synthesis of 4H-chromene via CSA@g-C_3_N_4_.


Entry	Catalyst	Catalyst (mg)	Solvent (1 mL)	Yield (%)^a^
1	CSA@g-C_3_N_4_	40	Ethanol	92
2	CSA@g-C_3_N_4_	30	Ethanol	84
3	CSA@g-C_3_N_4_	20	Ethanol	68
4	CSA@g-C_3_N_4_	10	Ethanol	56
5	CSA@g-C_3_N_4_	0	Ethanol	25
6	CSA@g-C_3_N_4_	40	CH_2_Cl_2_	58
7	CSA@g-C_3_N_4_	40	Glycerol	61
8	CSA@g-C_3_N_4_	40	CH_3_CN	69
9	CSA@g-C_3_N_4_	40	H_2_O	48
10	CSA@g-C_3_N_4_	40	CH_3_CO_2_Et	54
11	CSA@g-C_3_N_4_	40	THF	62
12	CSA@g-C_3_N_4_	40	DMF	76
13	CSA	40	Ethanol	71
14	g-C_3_N_4_	40	Ethanol	18

^a^Isolated Yields.

Table 3 compares the different catalysts of CSA@g-C_3_N_4_ that have been synthesized by different amounts of loading CSA on the surface of the g-C_3_N_4_ catalyst. Actually, 4H-chromenes synthesis was performed under the mentioned optimum conditions, where the only objective was searching for the optimum value of CSA for loading on the g-C_3_N_4_ surface. Ultimately, the results obtained from Table 3 illustrated that the maximum efficiency would be gained for conducting the reaction by using 2 g of CSA is loaded on the g-C_3_N_4_ surface.

**Table 3:** Optimization of the loaded CSA amount on the surface of g-C_3_N_4_.


CSA (g)	0.5	1	1.5	2	2.5
Yield (%)	63	76	88	92	92

Derived from the fascinating results, elucidating the efficient impact of the powerful catalyst of CSA@g-C_3_N_4_ on multicomponent reactions, it was decided to conduct an additional investigation by carrying out further reactions through different substituted salicylaldehyde with thiols under the expressed conditions. Table 4 discusses about reactions between salicylaldehydes derivatives and several thiophenols in the presence of CSA@g-C_3_N_4_ catalyst. In this regard, salicylaldehydes with bromo as well as methoxy substitutions reacted with thiophenols such as thiophenol, para-bromothiophenol, para-methylthiophenol, para-methoxythiophenol, para-chlorothiophenol, and 2-naphthalenethiol at the temperature of 60 °C for 2 to 4 h. In such reactions, 4H-chromene derivatives can be gained by yields of 74% to 95% via both electron-rich salicylaldehyde, for example, 3-methoxysalicylaldehyde and electron-deficient salicylaldehyde, for instance, 5-bromosalicylaldehyde. Moreover, the results indicate the high efficiencies for all three-component reactions; it should be noted that applying 3-methoxysalicylaldehyde obtained a bit less yield rather than salicylaldehyde and 5-bromosalicylaldehyde. With this respect, mentioned aromatic thiols delivered chromene significant yields subjected to mild reaction conditions. Additionally, 2-naphthalenethiol illustrates a lower efficiency compared to thiophenol and thiophenols with substitutions in para position. Not only electron-withdrawing but also electron-donating substituent on the thiophenol showed any distinction in the selectivity as well as the reactivity of the reaction.

**Table 4:** Multicomponent synthesis of various substituted 4H-chromene via CSA@g-C_3_N_4_ under optimum conditions.


Entry	Salicylaldehyde (1)	Thiol (2)	Yields (%)^a^
1		2a	95
2		2b	95
3		2c	92
4		2d	95
5		2e	88
6		2f	81
7		2a	95
8		2b	94
9		2c	91
10		2d	93
11		2e	90
12		2f	86
13		2a	92
14		2b	89
15		2c	94
16		2d	74
17		2e	85
18		2f	80

^a^Isolated yields.

In continuation of gaining a better perceiving of the three-component reaction of chromene synthesis, when different nucleophiles, including secondary aliphatic amines, reacts with salicylaldehyde and malononitrile under the same reaction conditions, the generalization of this benign would obviously be approved. Moreover, a three-component condensation of salicylaldehyde, malononitrile, plus secondary amines undergoing the resembling reaction conditions was conducted to serve a quick approach to benzopyrano[2,3-d]pyrimidine to emphasize the capacity of this environmentally friendly method. Table 5 explores the synthesizing 4H-chromeno[2,3-d]pyrimidines derivatives attained via three-component reaction between salicylaldehyde, 5-bromosalicylaldehyde, and 3-methylsalicylaldehyde with malononitrile and diverse amines by the assistance of 40 mg CSA@g-C_3_N_4_ catalyst in existence of 3 mL ethanol as solvent at 60 °C for 3 to 5 h. Likewise, secondary aliphatic amines, which include piperidine, 4-methylpiperidine, morpholine, pyrrolidine, and diethylamine experienced a three-component reaction, bringing about the promised product in excellent yields. This three-component reaction was readily and practically accomplished by exploiting the robust as-prepared catalyst at the temperature of 60 °C. The highest yield of 90% for producing 4H-chromeno[2,3-d]pyrimidine was acquired when salicylaldehyde reacted with piperidine and 4-methylpiperidine (Table 5, Entries 1 and 2). On the other side, a reaction between 3-methylsalicylaldehyde with pyrrolidine resulted in the lowest yield of 68% (Table 5, Entry 8).

**Table 5:** Green synthesis of 4H-chromeno[2,3-d]pyrimidines derivatives via three-component condensation of salicylaldehyde, secondary aliphatic amines, and malononitrile by using CSA@g-C_3_N_4_.


Entry	Amines	Salicylaldehyde	Yields (%)^a^
1		**1a**	90
2		**1a**	90
3		**1a**	88
4		**1a**	77
5		**1a**	71
6		**1b**	78
7		**1b**	74
8		**1b**	68
9		**1c**	81
10		**1c**	83
11		**1c**	76

^a^Isolated yields.

To evaluate the catalytic performance of CSA@g-C_3_N_4_ nanocomposite, a comparison table (Table 6) from previously reported data is represented, which consists of the parameters, such as catalyst, yields, reaction time, and reaction conditions in synthesis of 4H-chromenes. The results depict that several drawbacks exist in previously reported methodologies, including high-price catalysts, non-reusable catalysts, harsh conditions for catalyst synthesizing, challenging separation, long reaction time, and low yield. As a whole, the present protocol shows considerable plus points over some of the other former reported methods owing to applying CSA@g-C_3_N_4_ as the catalyst.

**Table 6:** Comparative catalytic activity of CSA@g-C3N4 with other reported catalysts in synthesis of 4H-chromenes and 4H-chromeno[2,3-d]pyrimidines derivatives.

Entry	Catalyst	Conditions	Time	Yield (%)	References
1	[Bmim]BF_4_	EtOH, RT	30 min	80	^91^
2	Sodium formate	EtOH, RT	12 h	83	^92^
3	Piperazine-Amberlyst®15	EtOH, 50 °C	8 h	80	^93^
4	Brønsted acid ILs	Solvent-free, RT	14 min	93	^94^
5	SBA-IM-NH2	Ultrasonic, RT	10 min	80	^95^
6	Silica-bonded N-propylpiperazine sodium n-propionate	Solvent-free, RT	6 h	85	^96^
7	TBBDA	EtOH, RT	24 h	90	^97^
8	Ag@TEOS	EtOH, 60 °C	45 min	93	^98^
9	CSA@g-C_3_N_4_	EtOH, 60 °C	2 h	92	This work^a^
10	CSA@g-C_3_N_4_	EtOH, 60 °C	3 h	90	This work^b^

^a^Synthesis of 4H-chromenes.

^b^Synthesis of 4H-chromeno[2,3-d]pyrimidines.

### Reusability of CSA@g-C_3_N_4_

**Figure 7:**
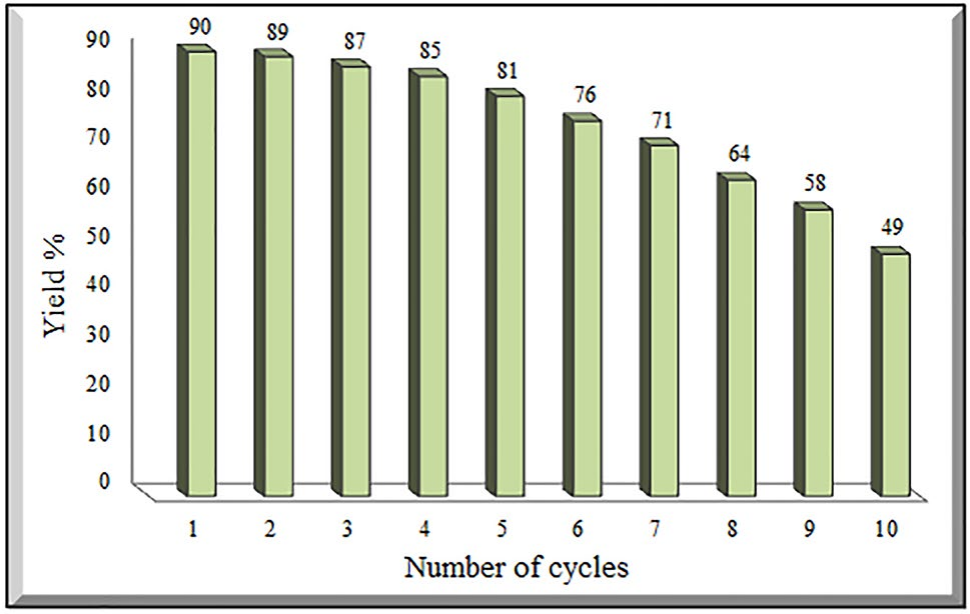
Recyclability of CSA@g-C_3_N_4_ for the synthesis of 4H-chromenes derivatives under optimum condition.

More notably, recyclability is of substantial importance in provisioning environmental sustainability together with economic pursuits. With respect to tackling this dilemma, the reusability of CSA@g-C_3_N_4_ for assessing its stability was checked in the direct synthesis of 4H-chromenes derivatives (Fig. 7). To recover the catalyst, it was separated through centrifuge first of all, then washed by warm ethyl acetate and ethanol in two steps, and finally, dried at room temperature to exploited again in chemical reactions. Moreover, the reusability and performance of the catalyst were assessed by executing several reactions in ten stages, based on the model reaction, utilizing thiophenol, malononitrile, and salicylaldehyde in attendance of solvent of ethanol and 40 mg of catalyst, results can be seen in Fig. 7. At least four times reusing caused a notable drop neither in catalytic activity nor in efficiency of the CSA@g-C_3_N_4_ catalyst, demonstrating the recycling capacity of this catalyst. It can be stated that almost from the fifth stage, the reaction efficiency gradually declines. In reviewing the results, the catalyst nature satisfactorily was not shifted throughout the recycling reactions. The elemental mapping of CSA@g-C_3_N_4_ and the EDS analysis of reused catalyst after ten runs were placed in the supporting information (see details in the Figure S3).

## Experimental section

### Materials and equipment

The entire chemicals were procured from the distinguished companies namely Merck and Aldrich while possessing high purities over 99%. The development of reactions were scrutinized via Thin-layer chromatography (TLC) in which silica gel 60 F254 was employed to immobilize the aluminum plates, visualized by UV illumination, afterwards. To evaluate the nano-catalyst traits, various procedures, for instance FT-IR, XRD, TGA, SEM, EDS, TEM added to BET were deployed. FT-IR 8400 manufactured by Shimadzu company was operated to record Fourier transform infrared spectroscopy (FT-IR) with a domain ranged from 400 to 4000 cm^−1^ for packed powder plates. The X-Ray diffraction (XRD) patterns attributed to the catalysts were gained from the AG D5000 tool (Siemens, Germany) to discern crystalline construction using Cu-Kα (λ = 1.54° A) as emission source. A scanning electron microscope (SEM) was utilized at 10 kV to determine the particle size of as-prepared catalyst. A scanning electron microscope apparatus (BAL-TEC SCD 005) was exploited. A helpful instrument of Energy-dispersive X-ray spectroscopy (EDS) was appointed to inspect the elemental distribution of synthesized catalyst implemented on an equipment model of MRIA3 TESCAN-XMU (Czechia). TEM images were recorded on a Philips EM 208S transmission electron microscope through the accelerating voltage adjusted to 100 kV. TGA was carried out to approve the reliability of catalyst synthesis as well as the thermal stability, which conducts a STA504 instrument at the temperature span from 0 to 800 °C with the heating rate value of 5 °C min^−1^ subjected to air atmosphere. A BELSORP Mini II nitrogen adsorption apparatus was a proper methodology to calculate the nitrogen adsorption–desorption isotherm at 77 K with respect to the Brunauer–Emmett–Teller (BET) surface area (*S*_BET_) accompanying the pore factors of representative samples while degassing treatment was performed at the temperature of 200 °C. Additionally, pore size distribution was measured according to the N_2_ adsorption isotherm through applying the Barrett-Joyner-Halenda (BJH) computational procedure based on the Halsey equation. The temperature-programmed desorption of ammonia (NH_3_-TPD) procedure was conducted to assess the acidic characterization of the samples via an NS-93 (NanoSORD) model with a temperature span from 100 to 600 °C.

### Preparation of g-C_3_N_4_

However, there are diverse methodologies for synthesizing g-C_3_N_4_, hence, a simple protocol along with cost-effective precursors are accounted as two deciding parameters which were considered in this work to prepare nano-catalyst. Subsequently, melamine by the value of 10 g was transferred to a furnace using a covered steel crucible to be subjected to 550 °C heating at a 5 °C/min heating rate for a time period of 3 h. After cooling off to room temperature, carbon nitride (g-C_3_N_4_) yellow powder was gained. Eventually, calcination of melamine would be completed. The bulk carbon nitride was conveyed to a furnace with a temperature set point of 550 °C for 2 h to deliver nano-scale particles of graphitic carbon nitride.

### Synthesis of CSA@g-C_3_N_4_

A round-bottom flask was chosen to hold a mixture of 25 ml dry DMF, 2 g CSA, and 2 g g-C_3_N_4_ to be heated up to 100 °C in a period of 12 h. Then, the entire acquired mixture was allowed to be cooled to room temperature prior to adding water and centrifuging. Consequently, CSA@g-C_3_N_4_ was reached after washing the solid multiple times via ethyl acetate and getting dried exposure to vacuum conditions at the temperature of 80 °C for 4 h.

### General strategy for the preparation of 4H-chromenes derivatives by using CSA@g-C_3_N_4_

A 5-mL balloon was deployed to carry 1 mL ethanol, 1 mmol of salicylaldehyde, 1 mmol of malononitrile, 1 mmol of the nucleophile, and 40 mg of CSA@g-C_3_N_4_, which were stirred for 2 h at the temperature of 60 °C. TLC detected the endpoint of the reaction, then 5 mL of water was poured during stirring, and the residue was gathered via filtration, afterward. The acquired solids or viscous liquids were washed by water, clarified through either column chromatography, or with the assistance of recrystallization utilizing diethyl ether or ethanol to provide pure produce.

### General protocol for the production of 4H-chromeno[2,3-d]pyrimidines via CSA@g-C_3_N_4_

In a 5-mL balloon equipped with a magnetic agitator, which consists of 3 mL of ethanol, salicylaldehyde, malononitrile, and secondary amines by the volume of 2, 1, and 1 mmol, respectively, were added, mixed at the temperature of 60 °C for 3 to 5 h. Additionally, TLC was operated as a helpful methodology to track the progression of the reaction, the catalyst was also separated by way of centrifuging subsequent to completely performing the reaction. In the end, to purify the products, ethanol, methanol, ethyl acetate, or tetrahydrofuran were found to be appropriate solvents.

## Conclusions

Novel catalyst of CSA@g-C_3_N_4_ introduced in this study embodies glaring traits such as high stability as well as non-toxicity accounted as a prime candidate for synthesizing chromenes through three-component reactions consisting of salicylaldehyde, thiophenol, and malononitrile. Incorporation of camphorsulfonic acid (CSA) into g-C_3_N_4_ is set to be a constructive and highly practical strategy for enabling immense catalytic activity along with providing simplicity, cost-effectiveness, and time-saving for conducting the process. The new organocatalyst of CSA@g-C_3_N_4_ was structured from low-price as well as straightforward substrates while removing metal to chase the goal of becoming a benign protocol through a mild organic transformation which is currently placed on the top dominant trends of both industry and academia. It was comprehended that the acidification of g-C_3_N_4_ brings about high yields in comparison with pure g-C_3_N_4_, entailing a facile purification after a mild and rapid reaction. The novel heterogeneous catalyst was identified via several methods, including FT-IR, XRD, SEM, EDS, TEM, TGA, and BET. Additionally, this catalyst is recyclable after executing at least ten reaction cycles without any drop in catalytic performance, which can reach the development of a sustainable process.

### Supplementary Information

Supplementary Information.

## Data Availability

The datasets used and/or analysed during the current study available from the corresponding author on reasonable request.
