# Supplementary material for: CSA@g-C3N4 as a novel, robust and efficient catalyst with excellent performance for the synthesis of 4H-chromenes derivatives
Source: Sci Rep. 2023 Nov 3;13:18961. doi: 10.1038/s41598-023-46122-y (PMC10624862; doi:10.1038/s41598-023-46122-y)
Supplement: Supplementary file 1 — Supplementary Information. [file 41598_2023_46122_MOESM1_ESM.docx]

**CSA@g-C_3_N_4_ as a novel, robust and efficient catalyst with excellent performance for the synthesis of 4H-chromenes derivatives**

*Saber Hosseini^1^*, Najmedin Azizi^1^**

^1^ Chemistry and Chemical Engineering Research Center of Iran, P.O. Box 14335-186, Tehran, Iran.

Email: [saber.hosseini89@gmail.com](mailto:saber.hosseini89@gmail.com), [azizi@ccerci.ac.ir](mailto:azizi@ccerci.ac.ir)


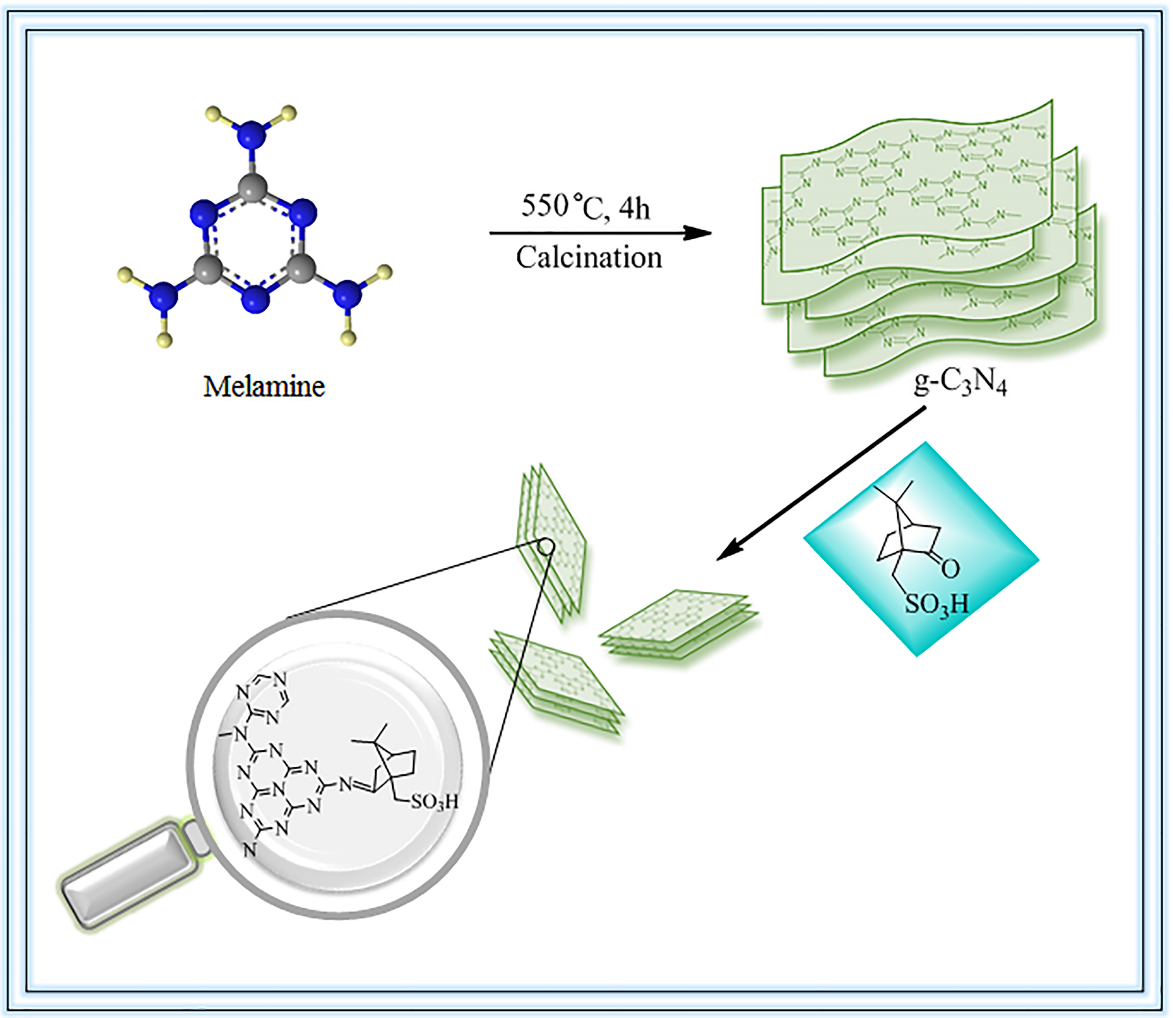


**Figure S1.** Preparation of CSA@g-C_3_N_4_ nano-catalyst.


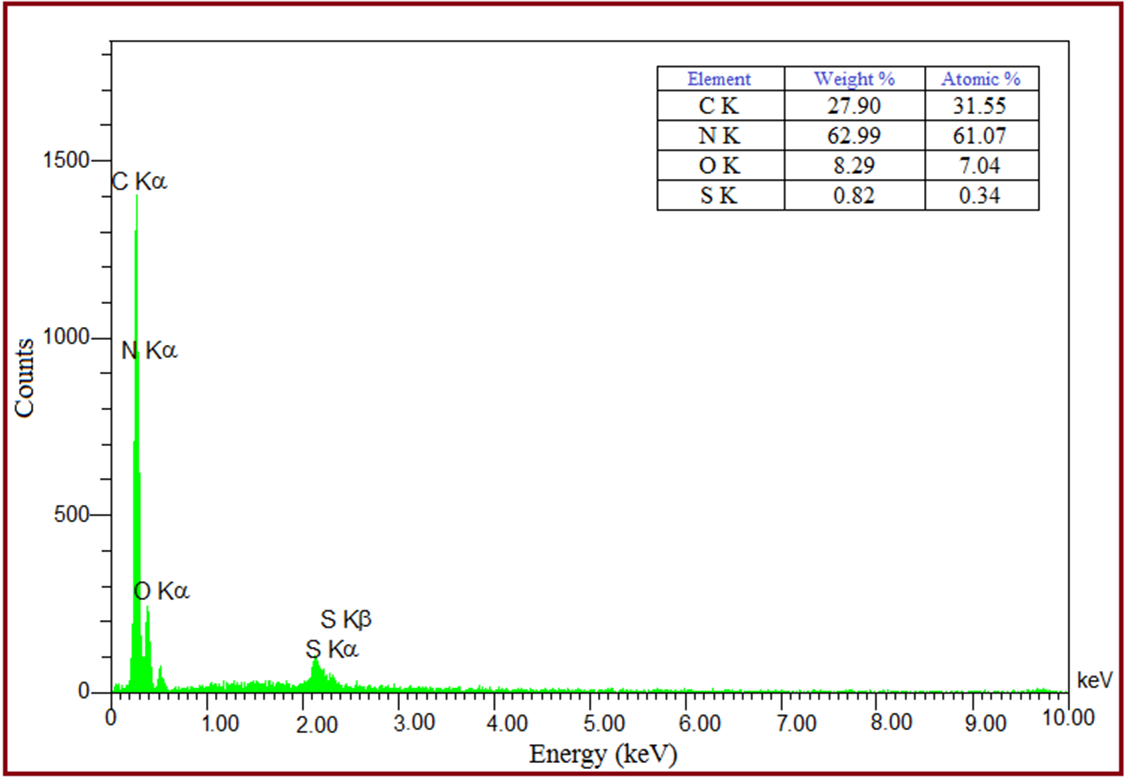


**Figure S2.** EDS analysis of CSA@g-C_3_N_4_.


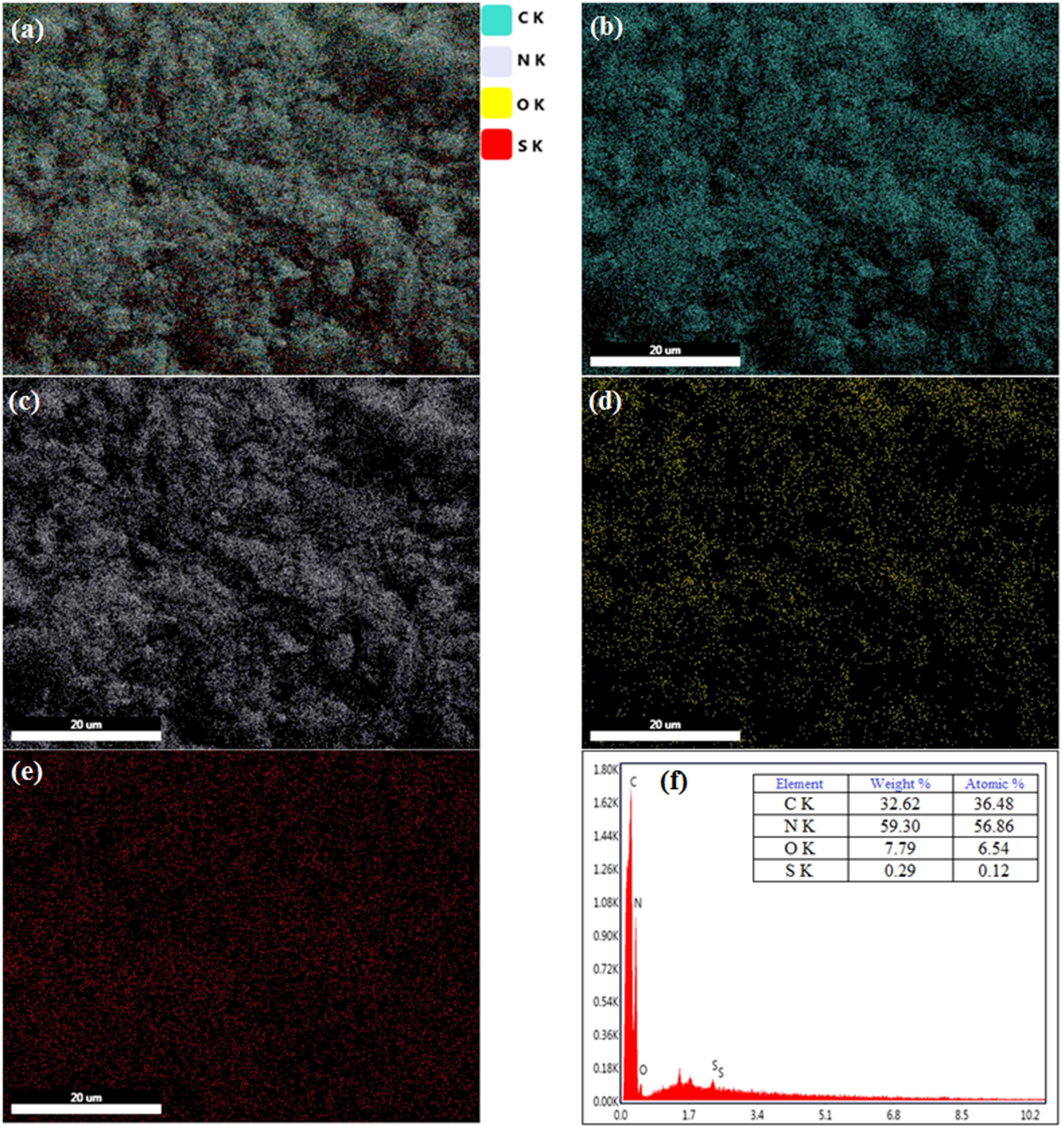


**Figure S3.** (a) The elemental mapping of CSA@g-C_3_N_4_, (b) carbon, (c) nitrogen, (d) oxygen, (e) sulfur, and (f) The EDS analysis of reused catalyst after ten runs.

**Table S1.** BET surface areas and pore parameters of pure g-C_3_N_4_ and CSA@g-C_3_N_4_

| **catalyst** | **S_BET_ (m^2^/g)** | **Pore volume^a^**  **(cm^3^/g)** | **Pore size^b^ (nm)** | **Porosity^d^ (%)** |
| --- | --- | --- | --- | --- |
| Pure g-C_3_N_4_ | 10.358 | 0.023 | 8.979 | 4.74 |
| CSA@g-C_3_N_4_ | 31.808 | 0.184 | 23.123 | 28.48 |

^a^ BJH adsorption cumulative volume of pores

^b^ Average pore diameter, estimated using the adsorption branch of the isotherm and the BJH formula.

^C^ Based on pore volume and 2.16 g/m^3^ of g-C_3_N_4_ density. Porosity (%) = pore volume (cm^3^/g)/(pore volume (cm^3^/g) + catalyst volume without pore (cm^3^/ g)) × 100; catalyst volume without pore (cm^3^/g) = 1/density of the catalyst volume without pore.
